# Reduced Number of Transitional and Naive B Cells in Addition to Decreased BAFF Levels in Response to the T Cell Independent Immunogen Pneumovax^®^23

**DOI:** 10.1371/journal.pone.0152215

**Published:** 2016-03-31

**Authors:** Alena Roth, Stephanie Glaesener, Katharina Schütz, Almut Meyer-Bahlburg

**Affiliations:** Pediatric Pneumology, Allergy and Neonatology, Hannover Medical School, Hannover, Germany; Albany Medical College, UNITED STATES

## Abstract

Protective immunity against T cell independent (TI) antigens such as *Streptococcus pneumoniae* is characterized by antibody production of B cells induced by the combined activation of T cell independent type 1 and type 2 antigens in the absence of direct T cell help. In mice, the main players in TI immune responses have been well defined as marginal zone (MZ) B cells and B-1 cells. However, the existence of human equivalents to these B cell subsets and the nature of the human B cell compartment involved in the immune reaction remain elusive. We therefore analyzed the effect of a TI antigen on the B cell compartment through immunization of healthy individuals with the pneumococcal polysaccharide (PnPS)-based vaccine Pneumovax^®^23, and subsequent characterization of B cell subpopulations. Our data demonstrates a transient decrease of transitional and naïve B cells, with a concomitant increase of IgA^+^ but not IgM^+^ or IgG^+^ memory B cells and a predominant generation of PnPS-specific IgA^+^ producing plasma cells. No alterations could be detected in T cells, or proposed human B-1 and MZ B cell equivalents. Consistent with the idea of a TI immune response, antigen-specific memory responses could not be observed. Finally, BAFF, which is supposed to drive class switching to IgA, was unexpectedly found to be decreased in serum in response to Pneumovax^®^23. Our results demonstrate that a characteristic TI response induced by Pneumovax^®^23 is associated with distinct phenotypical and functional changes within the B cell compartment. Those modulations occur in the absence of any modulations of T cells and without the development of a specific memory response.

## Introduction

Immune responses against T cell independent (TI) antigens are characterized by B cell activation and by generation of antibody production without the requirement for MHC class II-restricted activation by helper T cells [1]. TI antigens can be divided into two classes, namely TI type 1 (TI-1) and type 2 (TI-2) antigens. TI-1 antigens are polyclonal B lymphocyte activators that elicit a mitogenic response regardless of antigen-specificity. They do not directly ligate with the BCR but utilize more unspecific receptors including Toll-like receptors (TLR), which can result in cytokine secretion in addition to immunoglobulin production [1, 2]. Unlike T cell dependent or TI-2 antigens, TI-1 antigens are also able to directly stimulate immature in addition to mature B cells. In contrast, TI-2 antigens are formed by large multivalent molecules with repetitive epitopes such as bacterial polysaccharides (PS) that can stimulate B cells by high avidity BCR crosslinking in the absence of direct cognate T cell help. Thereby, cytokines produced from other cells, including T, NK and B cells, increase the magnitude of the immune response.

Encapsulated bacteria such as *Streptococcus pneumoniae*, *Haemophilus influenzae*, or *Neisseria meningitidis* represent major TI antigens that constitute a combination of capsular PS (TI-2) antigens with several TLR ligands (TI-1) contained in their bacterial cell walls. The generation of specific Abs against these pathogens is of great relevance, in particular for groups at a higher risk for infections. Elderly people, young children, or immuno-compromised patients show an increased susceptibility to infections with encapsulated bacteria, which cause a high rate of morbidity and mortality among these individuals [3–5]. In particular, patients with primary immunodeficiency with a defective B cell function, most importantly the so-called common variable immunodeficiency (CVID), suffer from recurrent infections with encapsulated bacteria a fact that underscores the important role of B cells in the defense against these bacteria [6, 7]. However, the nature of the human B cell compartment involved in this immune reaction is barely characterized.

In mice, marginal zone (MZ) B cells and B-1 cells have been identified as the main players in TI immune responses, and both B cell subsets have been characterized in great detail [8, 9]. In humans, CD27^+^IgM^+^ memory B cells have been described as equivalents of MZ B cells in the peripheral blood and were found to be in charge of PnPS-specific Ab production [10–12]. Human B-1 cells were recently characterized in umbilical cord, and adult peripheral blood as CD19^+^CD27^+^CD20^+^CD70^-^CD69^-^CD43^+^ cells [13]. However, the role of proposed human counterparts of MZ B cells and B-1 cells regarding phenotype, formation, and specificity against TI antigens remains barely defined [14–17] and literature discusses both classifications high controversially [13, 18, 19].

In this study we therefore examine in great detail the effect of a TI immunogen on the B cell compartment, characterizing B cell subpopulations contributing to the TI-immune response in respect to phenotypical, and functional aspects. Our results show that Pneumovax^®^23 directly impacts the composition of the B cell compartment, whereby IgA plays a primary and characteristic role.

## Materials and Methods

### Study cohort

The sample for the study comprised 30 healthy volunteers (14 males, 16 females; age range from 19 to 63 years). Each volunteer provided information about previous illnesses, present health and medication as well as written consent. Exclusion criteria were any autoimmune disease, immunodeficiency or pneumococcal vaccination in the last 5 years. The study protocol was approved by the local ethics committee of Hanover Medical School and in accordance with the Declaration of Helsinki.

### Vaccination and specimen collection

All volunteers were vaccinated with one dose of Pneumovax^®^23, administered i.m. in the M. deltoideus. As an unconjugated 23-valent capsular polysaccharide vaccine, 0.5 ml of Pneumovax^®^23 contains 25 μl of the pneumococcal serotypes 1, 2, 3, 4, 5, 6B, 7F, 8, 9N, 9V, 10A, 11A, 12F, 14, 15B, 17F, 18C, 19F, 19A, 20, 22F, 23F and 33F.

Serum and blood samples (30 ml heparinized blood) were obtained pre-vaccination and on day 7, 14, 28, 42, and 90 post-vaccination and assessed for enumeration of Pneumovax^®^23-specific ASC and Ig concentration. Induced sputum for determination of secretory antibodies was obtained from nine volunteers on day 0, and 28. After pretreatment with 100 μl Salbutamol (Sultanol, GlaxoSmithKline, Munich), a 5.85% saline solution was inhaled over a period of 20 min. Expectorated sputum samples were collected separately from saliva, and supplemented with 10 μl/ml protease inhibitor (Thermo Scientific). Serum and sputum were stored in aliquots at -80°C until analyzed.

### Reagents and Abs

Pneumovax^®^23 for vaccination of healthy volunteers, and coating of ELISpot plates was obtained from Sanofi Pasteur MSD (Leimen, Germany). The following fluorochrome-conjugated mAB were used: Pe-Cy7/PerCP-Cy5.5-anti-CD19, APC-Cy7-anti-CD27, APC/PerCP-Cy5.5-anti-CD38, PacificBlue-anti-CD20, FITC-anti-CD43, APC-Cy7-anti-CD69, PE-anti-CD70, PacificBlue-anti-IgM, PE-anti-CD3, APC-Cy7-anti-CD5, APC-anti-CD4, APC-Cy7-anti-CD8, PerCP-Cy5.5-anti-CxCR5, FITC-anti-ICOS and PE-anti-PD-1 (BioLegend, San Diego, CA, USA); PE-anti-CD138 and PE-anti-IgA (Miltenyi Biotech, Auburn, CA, USA); APC-anti-CD27 (eBioscience, San Diego, USA); and PE-Cy7-anti-IgG (BD Pharmingen, San Diego, CA, USA). Live/Dead^®^ fixable stain and normal mouse serum (NMS) were obtained from LifeTechnologies (Darmstadt, Germany). For ELISA and ELISpot assays, the following mAbs and Kits were purchased: HRP-IgM, HRP-IgG and HRP-IgA (SouthernBiotech, Birmingham, AL, USA), VaccZyme Human Anti-PCP IgM, IgA or IgG EIA Kits (Binding Site, Birmingham, UK) and “Quantikine ELISA, Human BAFF/BLys/TNFSF13B Immunoassay” (R&D Systems, Wiesbaden, Germany). For the detection step of the ELISpot assays, 3-amino-p-ethylcarbazole substrate solution (AEC) was used, provided by Vector (Burlingame, USA).

### Cell preparation, flow cytometric analysis and cell sorting

PBMC, isolated by Biocoll density-gradient centrifugation, were resuspended in staining buffer (2% FCS in PBS), followed by an additional 50 μg/ml NMS. Eight-colour immunophenotyping was performed on a three-laser FACSCantoII (BD Biosciences, Heidelberg, Germany), for three different staining panels, then analyzed with FlowJo Software versions 9.4/10.0.8r1 (Ashland, OR, USA). B cells were identified as CD19^+^ cells. Total B cells were separated into early transitional 1 (T1; CD27^-^CD38^hi^CD24^hi^), late transitional 2 (T2; CD27^-^CD38^int^CD24^hi^), mature naïve B cells (CD27^-^CD38^lo^CD24^lo^), plasma cells (PC; CD27^++^CD38^hi^CD138^hi^), and plasma blasts (PB; CD27^++^CD38^hi^CD138^lo^) (S1 Fig). Total CD27^++^ PB/PC and CD27^+^ memory B cells were further differentiated into IgM^+^, IgA^+^ and IgG^+^ cells. B-1 cells were gated as CD19^+^CD27^+^CD20^+^ CD70^-^CD69^-^CD43^+^ cells. Follicular helper T cells were characterized as CD4^+^CXCR5^+^PD-1^+^ cells. Exclusion of dead cells was performed by Live/Dead^®^ fixable stain, according to manufacturer’s recommendations. Compensation controls were set up with compensation beads, and gates were positioned based on fluorescence-minus-one controls (FMO). For isolation of B cell subsets, the B cells were enriched by magnetic cell enrichment (EasySep; StemCell Technologies, Cologne, Germany), stained with PECy7-anti-CD19/ APC-anti-CD27 and sorted using the FACSAria cell sorter (BD Bioscience).

### Cell cycle analysis

Cell cycle analysis was determined from isolated B cells after surface staining, and fixation. Percentage of cells in each phase of cell cycle was assessed by staining with DAPI, and PyroninY. Data was analyzed using FlowJo software.

### ELISpot

96-well microtiter plates (MultiScreen HTS, Merck, Darmstadt) were coated with 10 μg/ml Pneumovax^®^23 overnight at 4°C followed by the addition of 2% dry-skimmed milk in PBS to block unspecific binding. Subsequently, PBMC were adjusted to a concentration of 5x10^5^ cells/200 μl RPMI, and left at 37°C overnight. For immunoglobulin detection HRP-conjugated antibodies (anti-IgM 1:200, anti-IgA 1:100, anti-IgG 1:100 in PBS) were applied. After one hour of incubation, 50 μl/well of AEC substrate solution was added. Staining reaction was stopped with tap water, and plates were dried overnight.

Spots were counted with the A.EL.VIS ELISpot reader (Hannover, Germany), and results were expressed as IgM, IgA or IgG ASC per ml blood.

### ELISA

For quantification of pneumococcal capsular polysaccharide (PnPS)-specific antibodies in serum and sputum, IgM, IgA and IgG were measured by ELISA. Serial dilutions were titrated in order to determine optimal antibody dilutions. Sputum from 9 volunteers was prepared using the VaccZyme Human Anti-PCP IgM, IgA, or IgG EIA Kits (Binding Site, Birmingham, UK) according to the manufacturer’s instructions with IgG portions predefined by Binding Site as mg/L and IgM and IgA as U/ml. For ELISA from serum samples, Pneumovax^®^23 was absorbed onto ELISA plates at a 1:100 dilution at 4°C overnight. Following one hour of blocking with 2% BSA in PBS, serum samples (1:5), and sputum (undiluted) were incubated for another hour at room temperature. Bound antibodies were detected by adding 50 μl HRP-conjugated mAb per well (anti-IgM 1:2000, anti-IgA 1:6000, anti-IgG 1:6000), diluted in blocking buffer, and again incubated for 1 hour at RT. Staining reaction was initiated by 50 μl of substrate solution, and stopped by the addition of H_2_SO_4_. ODs were obtained at 450 nm on a microplate reader (Synergy 2, BioTek, Winooski, US). Cell wall polysaccharides (CWPS) blocking agents were not used in the procedure since it was recently shown by Schutz et al. that CWPS, irrespective of the CWPS concentration added, did not have any significant effect on the overall PnPS-specific ELISA outcome [20].

The Quantikine ELISA kit for detection of BAFF was used according to the manufacturer’s recommendations, and each serum sample was tested in duplicates. Specific absorbance was measured at 450 and 560 nm, and OD was quantified by a Glomax Reader (Promega, Fitchburg, WI, USA). Analysis was performed with GraphPad Prism (San Diego, CA, USA).

### Statistical analysis

Statistical differences were determined using the Student’s t-test for independent samples and One-way ANOVA, with Bonferroni post-test using GraphPad Prism. Differences were considered statistically significant for p <0.05.

## Results

### Increase of IgA^+^ but not IgM^+^ or IgG^+^ memory B cells in response to Pneumovax^®^23

**Fig 1:**
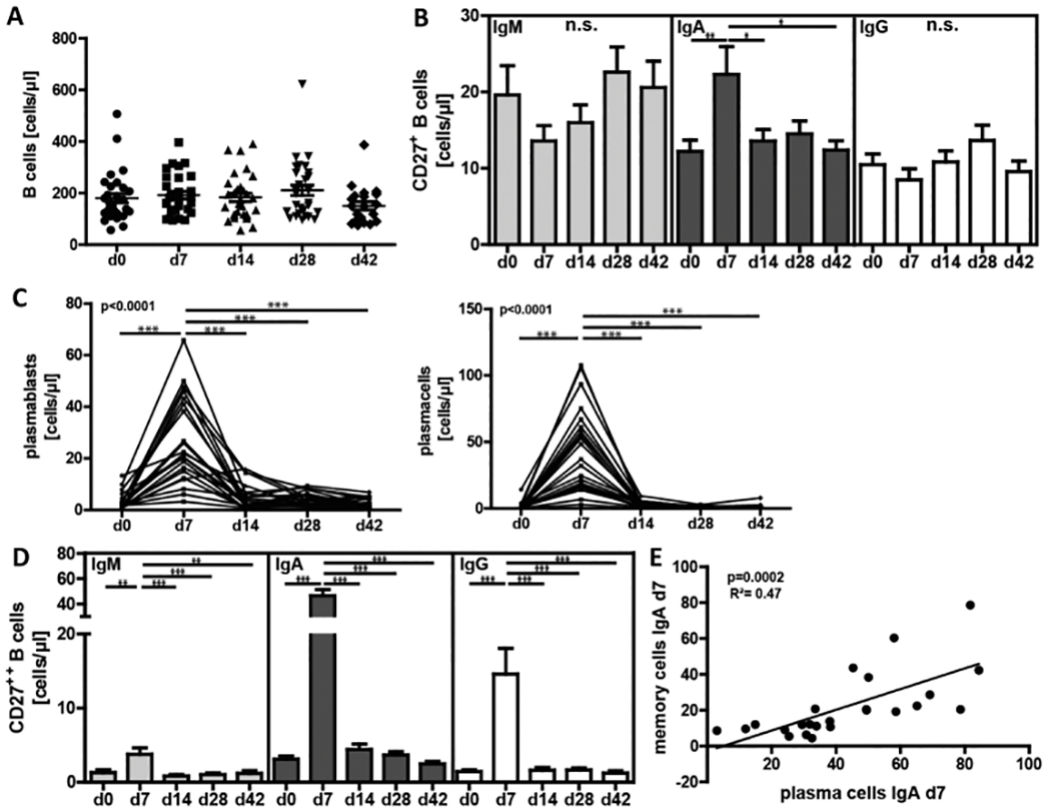
Increase in IgA^+^ memory B cells and plasma blasts/plasma cells. Absolute numbers of (**A**) total B cells, (**B**) IgM^+^, IgA^+^ and IgM^+^ CD27^+^ memory B cells, (**C**) CD27^++^CD38^hi^CD138^lo^ plasma blasts and CD27^++^CD38^hi^CD138^hi^ plasma cells and (**D**) IgM^+^, IgA^+^ and IgG^+^ CD27^++^ cells at indicated intervals after vaccination. (**E**) Positive correlation between IgA^+^ memory B cells and plasma cells at day 7. Data are shown from thirteen independently performed experiments per time point with n = 21. Mean values ± SEM are indicated for each time point; (*p<0.05, **p<0.01, ***p<0.001).

To analyze the effect of a TI-immune response on the B cell compartment in healthy individuals we used the pneumococcal polysaccharide-based vaccine Pneumovax^®^23. A phenotypical characterization of B cell subpopulations was performed by flow cytometry before (day 0) and on day 7, 14, 28, and 42 post-immunization. B lymphocytes were defined as CD19^+^ cells. They can be further differentiated into CD27^-^ naïve B cells or CD27^++^ plasma blasts (PB)/ plasma cells (PC) and CD27^+^ memory B cells with their respective Ig surface expressions (S1 Fig). No significant changes in the number of total CD19^+^ B cells were detected over the duration of the study (Fig 1A).

Because MZ B cells in addition to B-1 cells are crucial for TI immune responses [9], and human CD27^+^IgM^+^ memory B cells have been previously described as MZ B cell equivalents in human peripheral blood [12], we were particularly interested in this B cell subset. Our analysis revealed no significant alterations of CD27^+^IgM^+^ and CD27^+^IgG^+^ B cells but showed instead a significant increase of CD27^+^IgA^+^ memory B cells on day 7 in relative (29±12% day 0, 50±32% day 7, 36±15% day 14; data not shown) and absolute numbers (12±5 cells/μl day 0, 22±14 cells/μl day 7, 14±8 cells/μl day 14) (Fig 1B).

As expected, we detected a highly significant increase of CD19^+^CD27^++^ cells (data not shown) which can be further differentiated into CD19^+^CD27^++^CD38^hi^CD138^lo^ plasma blasts (PB) and CD19^+^CD27^++^CD38^hi^CD138^hi^ plasma cells (PC) [21–23]. Both PB (1±1cell/μl day 0, 27±14 cells/μl day 7; p<0.0001) and PC (1±1 cell/μl day 0, 44±27 cells/μl day 7; p<0.0001) were highly significantly increased at day 7 post vaccination (Fig 1C).

We next analyzed surface expression of IgA, M and G on CD19^+^CD27^++^ B cells. Consistent with the increase in IgA^+^ memory B cells, the majority of CD19^+^CD27^++^ PB/PC on day 7 expressed IgA (46±21 cells/μl; Fig 1D) and only few IgM or IgG (4±2 and 15±11 cells/μl respectively). Notably, as we performed a surface stain, immunoglobulin expression was not found on every CD19^+^CD27^++^ cell. Furthermore, the number of CD27^+^IgA^+^ memory B cells directly correlated to CD19^+^CD27^++^IgA^+^ PB/PC (p = 0.0002) on day 7 (Fig 1E) in contrast to IgM^+^ and IgG^+^ memory B cells (data not shown).

By day 14, in addition to the rest of the study duration, plasma and memory B cell counts returned to pre-immunization levels. Thus, our data shows a predominant expression of IgA on memory B cells and PB/PC in peripheral blood in response to pneumococcal polysaccharides.

### Evaluation of CD19^+^CD27^+^CD20^+^ CD70^-^CD69^-^CD43^+^ ‘B-1’ B cells in response to Pneumovax^®^23

**Fig 2:**
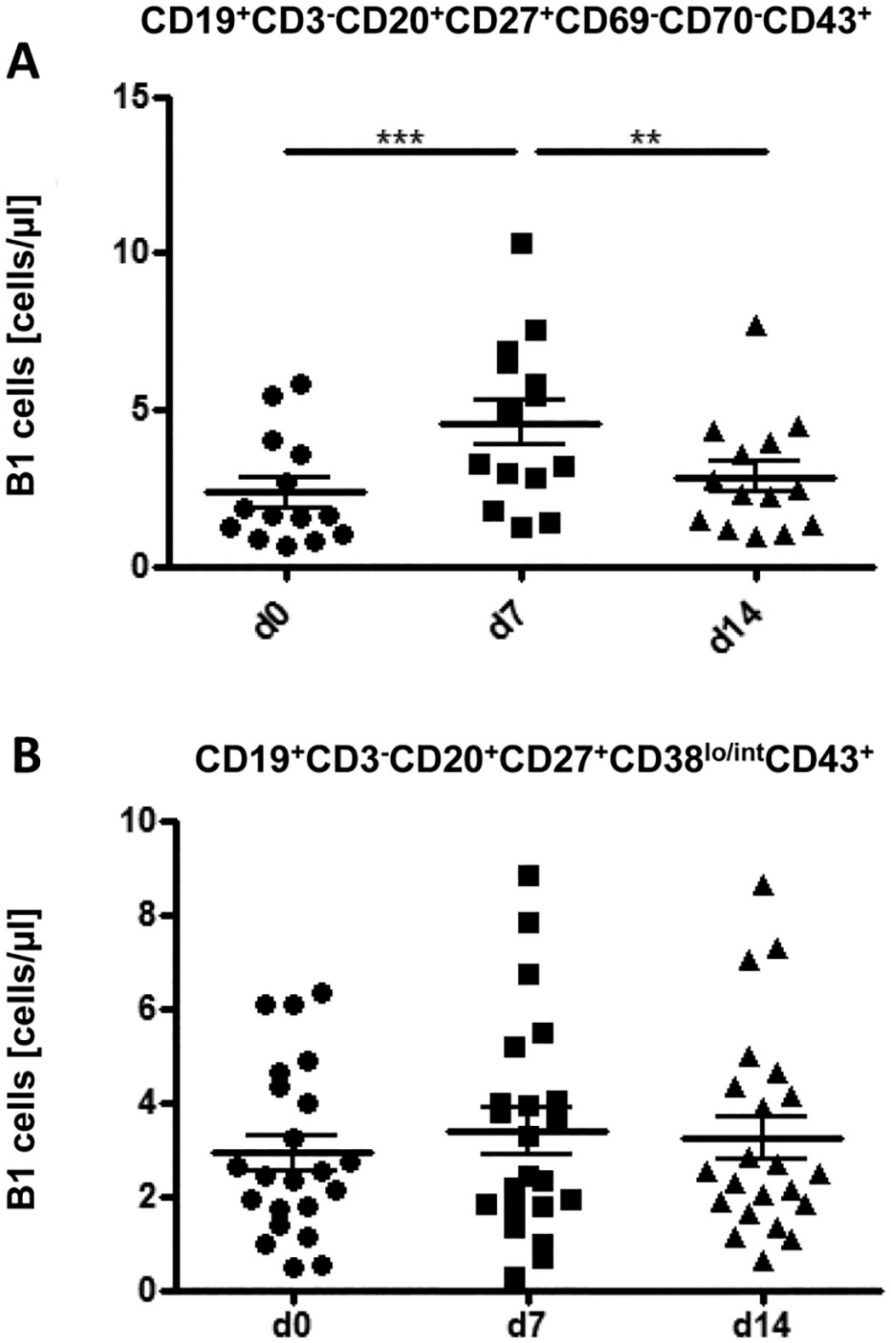
Involvement of proposed B-1 cells. (A) Absolute number of CD19^+^CD27^+^CD20^+^CD70^-^CD69^-^CD43^+^ B cells including CD27^++^ CD38^++^PC/PB after Pneumovax^®^23 vaccination evaluated by flow cytometry (n = 21). (**B**) Absolute number of CD19^+^CD20^+^CD27^+^CD38^lo/int^CD43^+^ B cells after exclusion of CD38^++^CD27^++^ PC/PB. Mean values ± SEM are indicated for each time point; (*p<0.05, ***p<0.001).

Besides MZ B cells, TI antigens cause the production of specific immunoglobulins by so called B-1 cells, as has been shown in mice [15]. Recently, human B-1 cells have been identified in peripheral blood of healthy adults [13], although this characterization remains controversial. We therefore investigated the involvement of recently described CD19^+^CD27^+^CD20^+^CD70^-^CD69^-^CD43^+^ cells (S2A Fig). Consistent with a previous study by Verbinnen et al. [24] our results show a highly significant increase of presumed B-1 cells from day 0 (1.2±0.5 cells/μl) to day 7 (2.3±1.3 cells/μl (p<0.0001), returning to pre-immunization levels on day 14 (Fig 2A). Regarding CD5 expression, CD5^-^ B-1 cells significantly increased, whereas CD5^+^ B-1 cells decreased slightly but not significantly (p = 0.001), consistent with a recent study [25] (data not shown). Because PC/PB cells can express CD43, more recent publications by Thomas Rothstein’s group suggest to exclude this population when analyzing human B-1 cells [26, 27]. Subsequently, B-1 cells were re-analyzed, thereby excluding CD38^++^CD27^++^ PC/PB (S2B Fig). Using this gating strategy, a significant increase in B-1 cells could no longer be detected (Fig 2B). Thus, no effect of Pneumovax^®^23 on proposed human B-1 B cells can be detected when this subset is carefully analyzed.

### Immunization with Pneumovax^®^23 impacts transitional and naïve B cell numbers

Because the total number of B cells did not change on day 7 post vaccination, despite a strong increase of memory B cells and PB/PC, other B cell subpopulations were expected to decrease correspondingly. We thus determined earlier B cell developmental stages, namely CD27^-^ naïve and transitional B cells. Based on CD24 and CD38 expression, they can be separated into early transitional 1 (T1; CD38^hi^CD24^hi^), late transitional 2 (T2; CD38^int^CD24^hi^) and mature naïve B cells (CD38^lo^CD24^lo^) (S1B Fig) [28].

**Fig 3:**
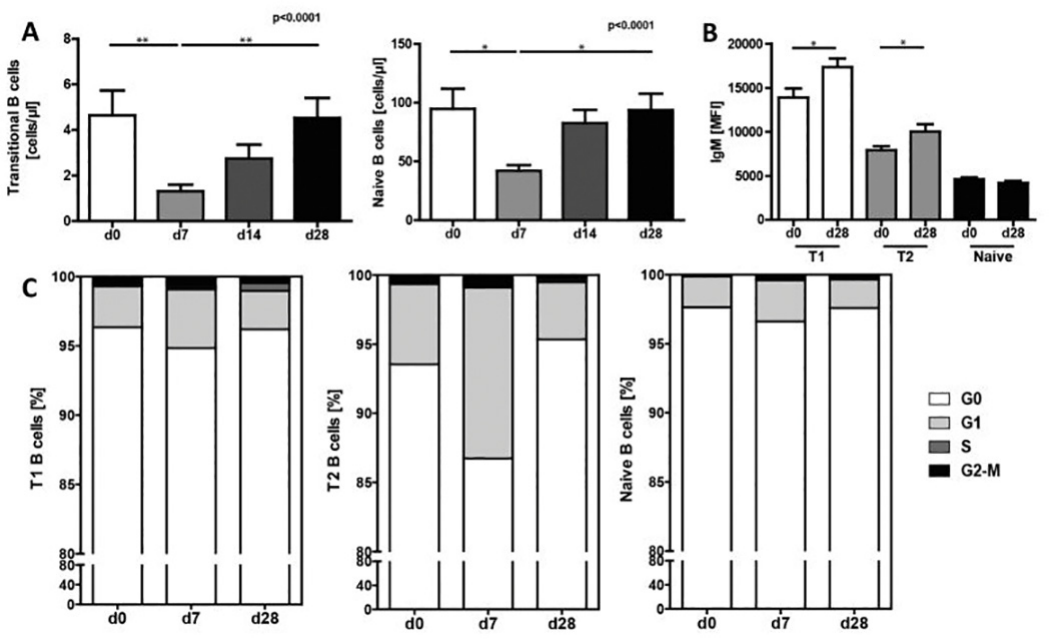
Immunization with Pneumovax^®^23 leads to transiently decreased numbers of transitional and naïve B cells. (**A**) Absolute numbers of transitional (T1+T2) and naïve B cells after immunization. Thirteen independently performed experiments with n = 21. (**B**) Expression levels of surface IgM on CD27^-^CD38^hi^CD24^hi^ T1, CD27^-^CD38^int^CD24^hi^ T2 and CD27^-^CD38^lo^CD24^lo^ naïve B cells compared between day 0 and 28. (**C**) Cell cycle analysis of transitional 1 and 2 and naive B cells (n = 9). Mean values ± SEM are indicated for each time point (*p<0.05 **p<0.01 ***p<0.001).

Our data demonstrates a significant decrease in the absolute cell count of the CD27^-^ B cell population from 116±48 cells/μl to 64±25 cells/μl one week after immunization, which returned to pre-immunization level by day 28 (p = 0.0009, S3 Fig). This decrease was caused by reduced numbers of both transitional and naïve B cells (Fig 3A).

The early decrease, followed by a subsequent raise of transitional and naïve B cells back to pre-immunization levels, suggests an increased rate of newly generated cells later in the immune response. Therefore, surface expression of immaturity markers was determined on day 0 pre-immunization to day 28 post-immunization. While we could not detect any changes in CD10 or CD21 expression (data not shown), T1 and T2 B cells expressed significantly higher levels of surface IgM on day 28 (p = 0.0005 T1 and p = 0.0034 T2) (Fig 3B). Enhanced IgM surface expression can indicate a more immature status of the transitional B cells as a consequence of a higher turn-over rate. This could be caused by proliferation of either peripheral, or bone marrow B cells. Due to availability, we then performed cell cycle analysis of the respective cell populations on day 0, 7, and 14 after immunization. While all three B cell subsets showed no significant change in the proportion of resting cells in G0 over time, T2 B cells tended to exhibit slightly more cells in G1 phase, although this was not significant (Fig 3C). We also analyzed kappa-deleting recombination excision circles (KREC) to determine recent proliferation in B cell subpopulations [29]. However, no increase of cell division could be detected in transitional or naïve B cells (data not shown).

We conclude from these results that Pneumovax^®^23 leads to an early consumption of transitional and naïve B cells, that are subsequently regenerated, most probably from bone marrow precursor B cells.

### T cells in peripheral blood are not affected by immunization with Pneumovax^®^23

To test a possible involvement of T cells in response to Pneumovax^®^23, we also analyzed the T cell compartment. We were particularly interested in the role of follicular helper T cells (T_FH_ cells) that directly interact with B cells and are crucial during the germinal center reaction for class-switching and affinity maturation [30]. In addition, T_FH_ cells have been shown to increase in response to influenza vaccination, a protein-based T-cell dependent immunogen [31]. We therefore determined the number of total CD4^+^ T cells and CD4^+^CXCR5^+^PD-1^+^ T_FH_ cells (S4A Fig). However, no significant changes in T cell subsets could be detected (S4B Fig), leading us to conclude that pneumococcal polysaccharides elicit a pure TI immune response.

### Pneumovax^®^23 immunization generates a strong PnPs-specific IgA response

**Fig 4:**
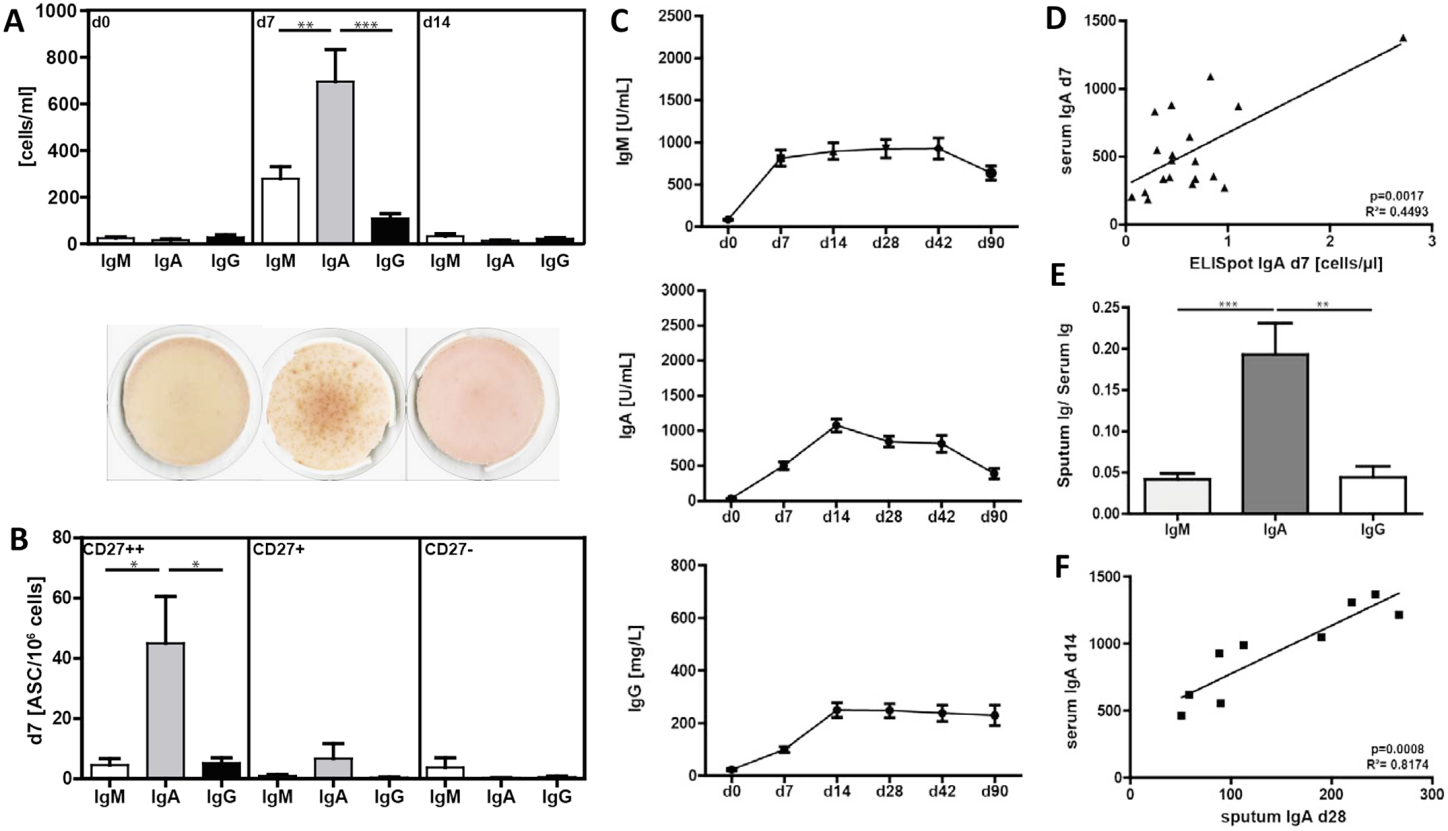
Kinetics of PnPS-specific IgA, IgM and IgG antibody secreting cells and antibodies. (**A**) Upper panel: PnPS-specific IgM, IgA and IgG as determined by ELISpot assay (n = 21). Lower panel: Representative ELISpot wells of one donor shown for IgA-producing PBMC on day 0, 7, and 14 after vaccination with Pneumovax^®^23. (**B**) PnPS-specific IgM, IgA and IgG ASC in sort-purified CD19^+^CD27^++^ plasma blasts/plasma cells, CD19^+^CD27^+^ memory B cells and CD19^+^CD27^-^ naïve B cells at day 7 post-vaccination. (**C**) Dynamics of PnPS-specific antibody responses in serum from day 0 to day 90 determined by ELISA (n = 30). (**D**) Positive correlation between PnPS-specific serum IgA on day 7 and IgA-producing cells. (**E**) Sputum/serum ratio of PnPs-specific Ig on day 28. (**F**) Positive correlation between PnPS-specific serum IgA on day 14 and IgA in sputum on day 28. Mean values ± SEM are indicated for each time point; (*p<0.05, **p<0.01, ***p<0.001).

We next tested the vaccine-induced antigen-specific B cell response in terms of frequency of PnPS-specific ASC, and antibody levels in serum and sputum. PnPS-specific IgA, IgM and IgG producing cells were determined by ELISpot assays. In agreement with previous studies [32], antigen-specific ASC were only detected on day 7 post vaccination (Fig 4A). We found significantly more PnPS-specific IgA ASC (696±414 cells/ml representing 64±30% of average total ASC) compared to PnPS-specific IgM (279±200 cells/ml) and IgG (109±74 cells/ml) ASC.

To further characterize PnPS-specific antibody-producing B cell subpopulations, we sort-purified CD27^++^ PB/PC, CD27^+^ memory and CD27^-^ naïve B cells on day 7 post immunization. As expected, PnPS-specific ASC were almost exclusively found within the PB/PC fraction (Fig 4B).

We also determined PnPS-specific Ig levels in the serum by ELISA. Because we previously could not detect an effect of CWPS blocking on overall PnPS-specific antibody titers pre- and post Pneumovax^®^23 vaccination [20], CWPS blocking was not used in this study. However, using this protocol, it is possible that cell wall polysaccharide antibodies are also measured. Consistent with results of Schutz et al. PnPS-specific IgM titers rose early on day 7, whereas IgA and IgG showed their peak level around day 14 post vaccination (Fig 4C). PnPS-specific IgA serum level directly correlated with the number of PnPS-specific IgA ASC on day 7 (p = 0.0017, R2 = 0.45) (Fig 4D).

*S*. *pneumoniae* can cause severe respiratory infections, and IgA is the major mucosal immunoglobulin. Thus, we also determined PnPS-specific antibody levels in induced sputum as first-line barrier of the lower and bronchial airways. Induced sputum on day 0 and 28 from nine of the immunized subjects was analyzed by ELISA. We found a significant increase of all three isotypes four weeks after vaccination. With a ratio of 0.19±9, IgA represented the highest sputum/serum proportion compared to 0.4±2 IgM and 0.4±3 IgG on day 28 (Fig 4E). Interestingly, IgA in sputum correlated highly significantly to PnPS-specific IgA in serum on day 14 (p = 0.0008, R2 = 0.82) (Fig 4F). No correlation could be detected among other Ig isotypes (data not shown).

### No specific memory response to pneumococcal polysaccharides in peripheral blood B cells

**Fig 5:**
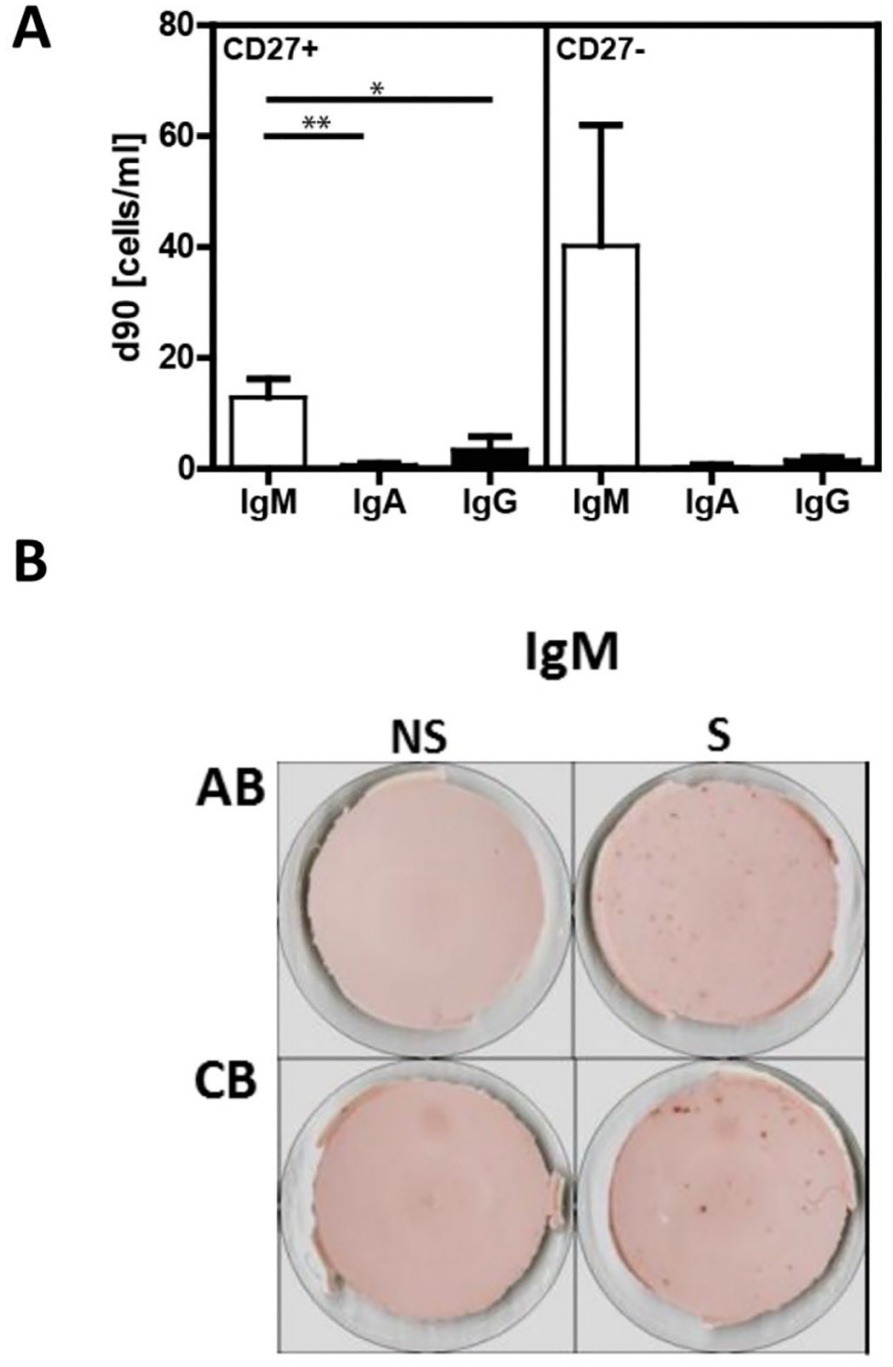
Vaccination with Pneumovax^®^23 does not generate a detectable memory response after 90 days. (**A**) Number of PnPS-specific antibody producing cells within CD27^+^ memory, and CD27^-^ naïve mature B cells purified by cell sorting 90 days after vaccination with Pneumovax^®^23. (**B**) Cord blood cells (CB), and PBMC of healthy adults not recently vaccinated with Pneumovax^®^23 (AB) were stimulated with a combination of IL-4/IL-21/anti-CD40/CpG/BAFF for 5 days, then examined for their PnPS-specific Ig production by ELISPOT. (*p<0.05, **p<0.01)

In order to determine the maintenance of immunological memory, we examined peripheral memory B cells by ELISpot assays for their capacity to produce PnPS-specific immunoglobulins three months after Pneumovax^®^23 immunization. Sort-purified CD27^+^ memory, and CD27^-^ B cells were stimulated with the TLR7/TLR8 agonist R848 combined with IL2 for 5 and 8 days [33]. Day 5 was found to be optimal for the detection of ASC, which were predominantly of the IgM-isotype (CD27^+^ p = 0.002; CD27^-^ p = 0.048) without significant difference between CD27^+^ and CD27^-^ B cells. Few antigen-specific IgG ASC could also be detected (Fig 5A). As a positive control, we determined tetanus toxoid in individuals that had received immunization with tetanus toxoid vaccine within the last 10 years. As expected, stimulation of PBMC resulted in the generation of tetanus toxoid-specific IgG ASC, but not IgA or IgM (data not shown).

Because PnPS-specific IgM producing cells were detected both in the CD27^-^ and CD27^+^ B cell fraction, this might be an unspecific response. To test this idea, we stimulated PBMC of healthy adults without previous immunization against pneumococci as well as cord blood cells. We then assessed the capability to produce PnPS-specific immunoglobulins. Again, cells were stimulated with either R848 and IL2 or a combination of IL-4, IL-21, anti-CD40, CpG and BAFF for 5 days. In both groups, PnPS-specific IgM ASC were found (Fig 5B), whereas no antigen-specific IgA or IgG ASC could be detected (data not shown). As for non-vaccinated adults, this immune response could be explained by previous contact with any type of pneumococcal serotypes. However, the production of PnPS-specific IgM from cord blood lymphocytes rather leads to consideration of an unspecific Ig response.

### Pneumovax^®^23 immunization results in transient reduction of BAFF serum levels

**Fig 6:**
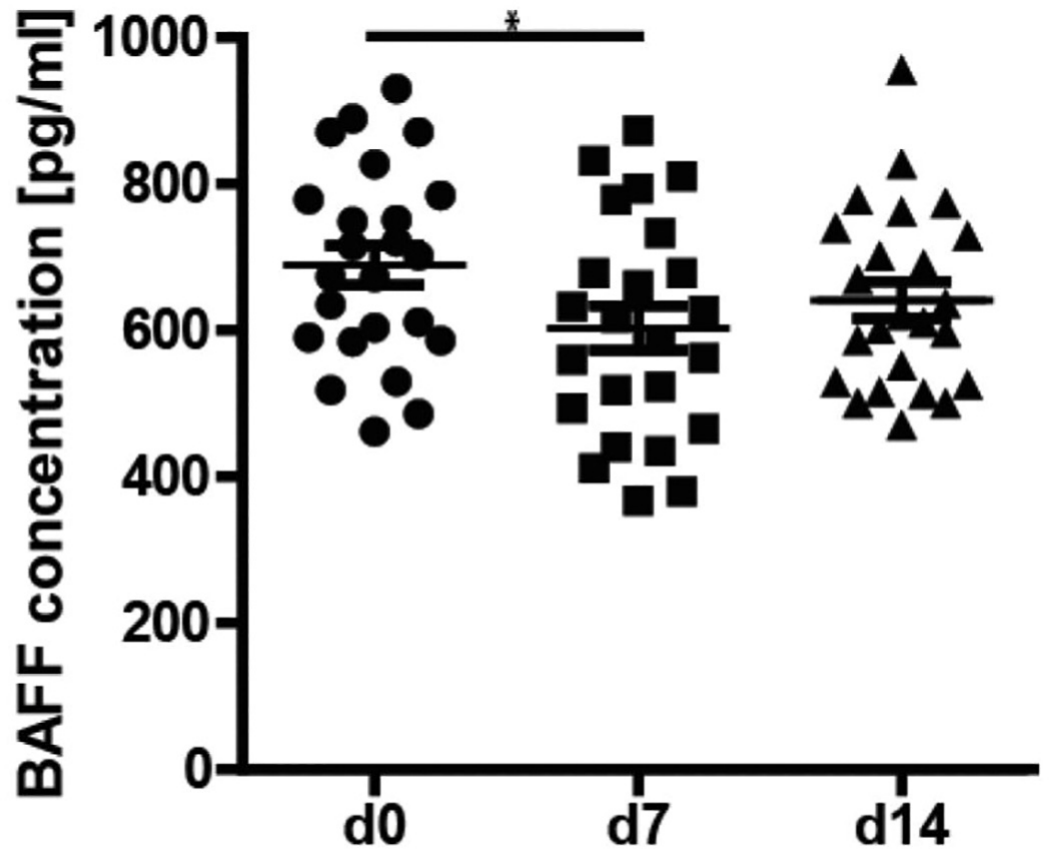
Transient decrease of serum BAFF levels. BAFF concentrations in the serum of 24 patients on day 0, 7 and 14 determined by ELISA. Mean values ± SEM are indicated for each time point; (*p<0.05).

B cell activating factor of the TNF family (BAFF) is an important B cell survival factor, and has been shown to be crucial for B cell development and differentiation in the periphery [34, 35]. In addition, BAFF has been suggested to be important for class-switching to IgA [36, 37]. Since we found a strong IgA response to Pneumovax^®^23, we determined serum BAFF levels by ELISA. Unexpectedly, our results demonstrate a slight, but significant decrease of BAFF levels on day 7 post-vaccination (689.5±132.7 pg/ml day 0; 603.1±149.5 pg/ml day 7) (Fig 6). Elevated BAFF levels turned back to pre-immunization levels by day 14 after immunization (641.6±123.7 pg/ml).

## Discussion

In the current study, we investigated the effect of a TI immune response on the human B cell compartment *in vivo* using Pneumovax^®^23 immunization. Isolated bacterial polysaccharides induce a TI-2 immune response. However, as recently demonstrated, the polysaccharide-based vaccine Pneumovax^®^23 contains contaminating TI-1 antigens such as TLR2 or TLR4 ligands that also have a co-stimulating influence on PnPS-specific B cells [1]. Thus, Pneumovax^®^23, combining TI-1 and TI-2 immunogens, can be considered as a model antigen for TI immune responses. Our data shows a strong IgA^+^ response, transient alterations in the composition of B cell subpopulation and decreased serum BAFF levels. No effect could be observed on recently proposed human CD19^+^CD27^+^CD20^+^ CD70^-^CD69^-^CD43^+^ B-1 cells or IgM^+^ memory B cells, presuming equivalents of MZ B cells.

Generally, infections can lead to activation of B cells, thereby impacting the composition of the B cell compartment, most importantly the generation of antibody-producing PC. The involvement of B cells depends on the pathogen, but only few studies exist demonstrating modulations of B cells for specific antigens. For TI responses, B-1 and MZ B cells have been well identified as main players in mice [9, 38]. Phenotypical characterizations of human equivalents of these B cell subpopulations have been described over the last years, but both characterizations remain highly controversial [13, 18, 19]. In addition, their involvement in TI responses was only barely investigated. Thus, in the current study, we aimed to directly test the effect on a TI antigen on proposed human B-1 and MZ B cells in peripheral blood.

Human B-1 cells were recently characterized as CD20^+^CD27^+^CD70^-^CD69^-^CD43^+^ B cells by Griffin et al. [13]. Using this classification, vaccination with Pneumovax^®^23 results in a significant increase of proposed B-1 cells on day 7 post-vaccination. Similar results were recently demonstrated by the group of Bossuyt [24]. However, in a follow-up study, this group finally concludes that CD20^+^CD27^+^CD43^+^ B cells might not be B-1 cells but rather plasma blasts or pre-plasma blasts as previously suggested [14, 18, 19]. Recently, the group by Rothstein recommended to exclude PC/PB when analyzing B-1 cells [27]. Consistent with these findings, after careful exclusion of CD27^++^CD38^++^ PC/PB from CD20^+^CD27^+^CD43^+^ B cells, an increase of proposed B-1 cells can no longer be detected.

In respect to MZ B cells, recent studies suggest CD27^+^IgM^+^ memory B cells as splenic MZ B cell equivalents in peripheral blood [11, 12]. The characterization is based on repertoire analyses of IgM^+^ memory B cells [39–41], and the finding that even in the absence of CD40-CD40L activation, CD27^+^IgM^+^ memory B cells can be found in peripheral blood, indicating that they comprise a distinct GC-independent B cell subset [42]. However, this characterization is not generally accepted and repeatedly discussed [17]. A more recent study Bagnara et al. performed high-throughput VH- sequencing on IgD^+^CD27^+^, IgD^-^CD27^+^ and IgD^-^CD27^-^ B cells from spleen and peripheral blood [43]. The study concludes that IgM memory and MZ B cells constitute two distinct entities. In our analysis, we could not observe significant alterations in the number of CD27^+^IgM^+^ memory cells. This is in contrast to another recent study, where immunization with Pneumovax^®^23 resulted in a significant decrease of IgM^+^ memory B cells [32]. Conversely, in this study healthy individuals where simultaneously vaccinated with Pneumovax^®^23 and tetanus toxoid, which may have affected the overall result.

Taken together, our results do not corroborate any alterations in proposed B-1 or MZ B cells within peripheral blood during a TI response. There are several possible explanations for this somewhat unexpected finding: 1. Peripheral blood does not represent the optimal sample material for analysis of B-1 and MZ B cells. Splenic MZ B cells or B-1 cells within the pleural or peritoneal cavity might have been affected, but these tissues are not easily accessible in healthy individuals2. Alternatively, antigen-specific B-1 and MZ B cells are present within the ASC pool following Pneumovax^®^23 immunization. However, due to their low numbers in peripheral blood they cannot be detected by the applied methods within the analyzed B cell subpopulations. 3. There are differences between mice and men, with human TI responses requiring different cell populations than those of mice. 4. The proposed phenotypical characterization of human B-1 and MZ B cells in peripheral blood is questionable, and does not identify the suggested cell populations.

Whereas proposed B-1 and MZ B cells were not affected after Pneumovax^®^23 immunization, alterations within other B cell subpopulations could be clearly observed, namely a decrease in transitional and naïve B cells as well as an increase in IgA^+^ memory B cells. Modulation of transitional and naïve B cells have been described in chronic HIV or Hepatitis C infection. In patients with chronic HIV infection, transitional and mature B cells are elevated [44], and can normalize after antiretroviral therapy [45, 46]. The disturbance of the B cell compartment is supposed to be responsible for increased susceptibility to invasive pneumococcal infection [46]. Similarly, chronic Hepatitis C virus infection is associated with an increase of transitional B cells [47].

Barely any data exists in respect to acute infections, and kinetic analyses of changes within the human B cell compartment caused by acute infections are severely limited due to experimental and ethical considerations. The problem can be bypassed using model antigens as we did in the current study with Pneumovax^®^23 as a characteristic TI antigen. The independence of T cells is underlined by the absence of any alteration in the T cell compartment, in particular T_FH_ cells, which have been found elevated in response to vaccination against influenza [31]. Instead, B cells are modulated in response to Pneumovax^®^23. Our results point towards a transient decrease of transitional and naïve B cells which could be caused by accelerated differentiation into PB/PC after activation. This theory is supported by the fact that overall B cell numbers do not change throughout the study period. Alternatively, inflammation induced by TLR ligands contained in the vaccine could alter B cell lymphopoiesis in the bone marrow resulting in mobilization and depletion of B cells in the bone marrow. This effect has been described in the mouse model following immunization with protein antigen in incomplete Freund’s as adjuvant [48]. The decrease of serum BAFF levels observed in our study may also contribute to the reduction in transitional and naïve mature B cells although it is probably too slight to explain this phenomenon.

Cell numbers turned back to the initial base-line value approximately on day 28. This was accompanied by enhanced expression of IgM but not CD10 on transitional B cells suggesting a wave of newly generated B cells later after vaccination and therefore implies enhanced B cell turnover. We could not detect significant changes in proliferation activity by cell cycle analysis or in the replication history measured by determination of KREC. This could be due to an insufficient small sample number. Alternatively, regeneration of the transitional and naïve B cell pool could be the result of enhanced migration from the bone marrow. In this case, bone marrow precursor B cells may have proliferated, and while we could not test this in our experiments, our study shows—to our knowledge for the first time—the direct impact of a TI immune response on transitional and naïve B cells in humans, and further demonstrates how different B cell subpopulations can be involved in distinct immune responses.

Though no modulations were found within IgM^+^ or IgG^+^ memory B cells, IgA^+^ memory B cells were significantly increased on day 7 post-vaccination. Overall, our study demonstrates a strong IgA response with elevated IgA^+^ memory B cells, predominant expression of IgA on CD27^++^ PB/PC B cells and PnPS-specific IgA ASC. Moreover, based on the ratio of IgA in induced sputum and serum, PnPS-specific IgA also seems to be over-represented in the lower airways. This is consistent with previous findings demonstrating significantly more antigen-specific IgA producing cells compared to IgM and IgG in response to Pneumovax^®^23 [32, 49]. A strong IgA response has been also shown in response to other bacterial polysaccharides including *Neisseria meningitidis* and *Hemophilus influenzae B* [50, 51].

When we tested for memory responses by stimulation of B cells on day 90 after vaccination, no IgA but only PnPS-specific IgM could be detected. However, PnPS-specific IgM production could likewise be detected in not-recently-vaccinated, healthy individuals and cord blood indicating the unspecific character of the response.

Despite the predominance of PnPS-specific IgA ASCs on day 7 post vaccinations, it must be kept in mind that PnPS-specific immunoglobulins of all isotypes are found in the serum over months and years and are mainly constituted by the IgG isotype. While this is probably produced by long-living PC in the bone marrow, the question remains open why in particular IgA^+^ cells are found in the peripheral blood early after vaccination. It is possible that distinct differentiation pathways of immune responses are involved in IgA vs IgG generation, and IgA is not only characteristic but also predictive for the strength of TI responses. In agreement with this idea Quinti et al. previously showed that very low total IgA serum levels are a prognostic factor for the development of pulmonary infectious complications in CVID patients [52]. Because IgA in serum and sputum directly correlate, antigen-specific IgA in induced sputum might also serve as a possible indicator of the strength of the immune response and respiratory protection.

Overall, our data suggests that IgA^+^ B cells and antibodies might therefore be characteristic for TI-driven immune responses. Even though B cells specific for certain pneumococcal serotypes such as PPS14 or PPS23 have been shown to be overrepresented by IgM^+^ memory cells [10, 53], they are not exclusively responsible for an overall TI immune response including TI-1 and TI-2 immunogens [54], but rather represent a nonspecific response, consistent with the idea of so-called natural IgM antibodies.

Because of the strong IgA response, and the demonstration of the importance of BAFF signaling for class switching to IgA, in particular in TI immune responses at mucosal surfaces [55], we determined BAFF serum levels before and after vaccination. Unexpectedly, we found a significant decrease of BAFF levels on day 7. In mice, splenic B cells BAFF has been shown to induce *in vitro* class switching to IgA. The effect of BAFF was mediated through both BAFF-R and TACI [56]. Similar results were demonstrated in human B cells. Litinskiy et al. demonstrated that stimulation with BAFF of human purified naïve B cells results in class switching to IgA in addition to IgG [57, 58]. This effect was independent of CD40 and mediated by dendritic cells (DC). Consistent with these results, mutations in the BAFF receptor TACI can cause CVID or selective IgA deficiency [58, 59]. Others investigated BAFF in the context of responses to bacterial capsular polysaccharides. One *in vivo* study demonstrated that BAFF injection increases the IgA response to Pneumovax^®^23 in mice [60]. *In vitro* it was shown that stimulation of DC with capsular polysaccharides results in BAFF induction [61]. Our data suggest, that soluble BAFF is utilized by differentiating PC, thereby supporting class switching to IgA. This results in decreased BAFF levels within the serum. However, further studies are required to prove this hypothesis.

In summary, our results demonstrate that a characteristic TI-response induced by Pneumovax^®^23 is associated with distinct phenotypical and functional changes within the B cell compartment in the absence of any modulations of T cells, or the development of a specific memory response.

## Supporting Information

S1 Fig Gating strategy of (A) plasma blasts (CD19^+^CD27^++^CD38^+^CD138^-^) and plasma cells (CD19^+^CD27^++^CD38^+^CD138^+^), (B) early transitional 1 (T1; CD27^-^CD38^hi^CD24^hi^), late transitional 2 (T2; CD27^-^CD38^int^CD24^hi^) and mature naïve B cells (CD27^-^CD38^lo^CD24^lo^) and (C) CD27^++^ plasma cells, CD27^+^ memory cells and CD27^-^ B cells with their respective IgM, IgG or IgA surface expression. Flow cytometry was performed on a three-laser FACSCantoII and analyzed with FlowJo Software 9.4.
(TIF)

S2 Fig (A) Gating strategy of CD19^+^CD3^-^CD20^+^CD27^+^ CD70^-^ CD69^-^CD43^+^ and (B) CD19^+^CD3^-^CD20^+^CD27^+^CD38^lo/int^CD43^+^ B-1 cells after exclusion of CD38^++^CD27^++^ PC/PB. Flow cytometry was performed on a three-laser FACSCantoII and analyzed with FlowJo Software 10.0.8r1.
(TIF)

S3 Fig Pneumovax^®^23 has a direct impact on the CD27^-^ B cell population. Absolute numbers of CD27^-^ B cells after immunization. Mean values ± SEM are indicated for each time point (*p<0.05 **p<0.01).
(TIF)

S4 Fig Vaccination with Pneumovax^®^23 does not affect T cells. (**A**) Gating strategy of CD4^+^CxCR5^+^PD-1^+^ T_FH_ cells with absolute numbers of (**B**) CD4^+^ T cells and (**C**) CD4^+^CxCR5^+^PD-1^+^ T_FH_ cells after immunization (n = 20).
(TIF)
