# Supplementary figures and images for: Reduced Number of Transitional and Naive B Cells in Addition to Decreased BAFF Levels in Response to the T Cell Independent Immunogen Pneumovax®23
Source: PLoS One. 2016 Mar 31;11(3):e0152215. doi: 10.1371/journal.pone.0152215 (PMC4816312; doi:10.1371/journal.pone.0152215)

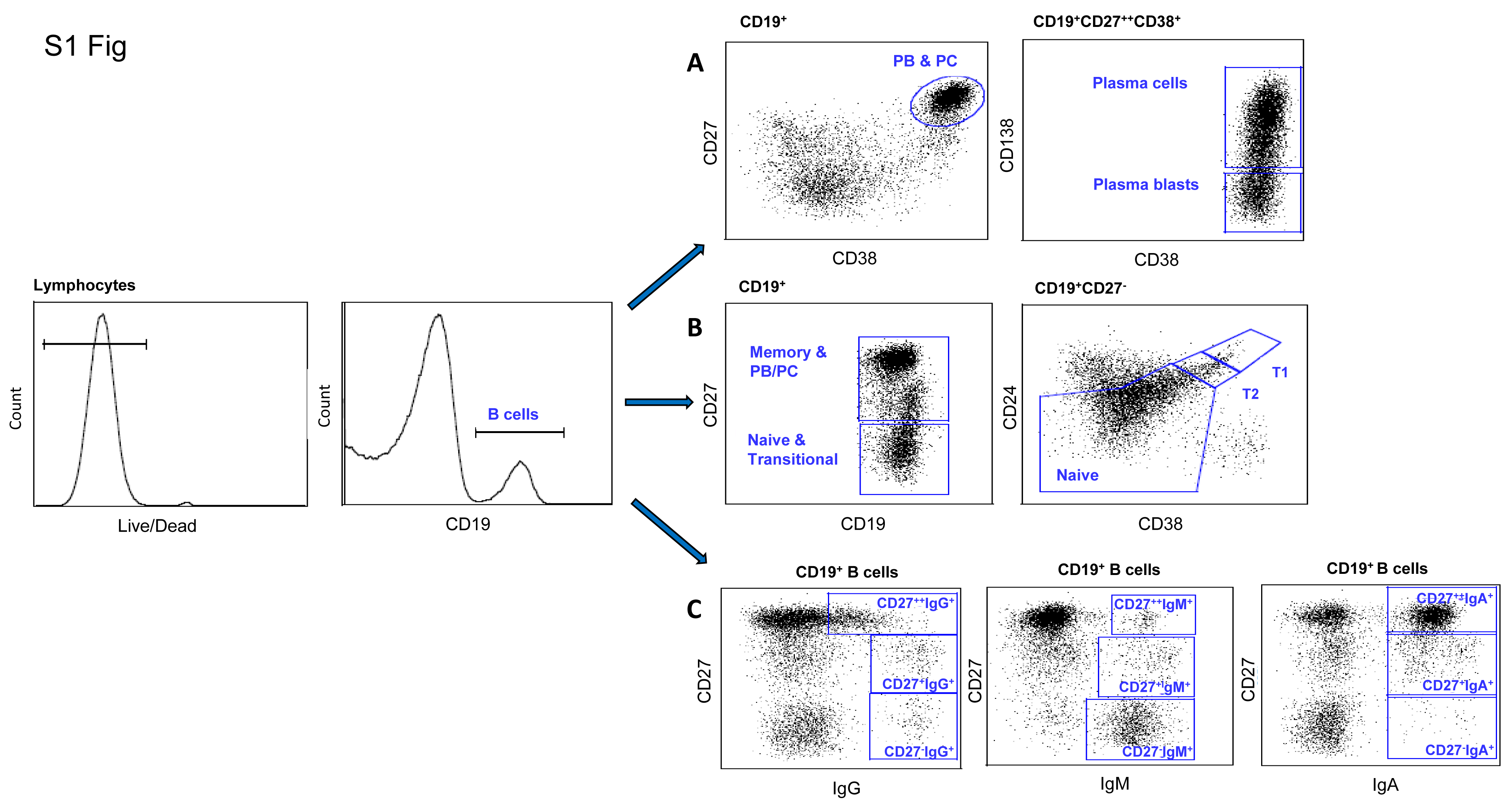

Supplement: S1 Fig — Flow cytometry was performed on a three-laser FACSCantoII and analyzed with FlowJo Software 9.4. (TIF) [file pone.0152215.s001.tif]

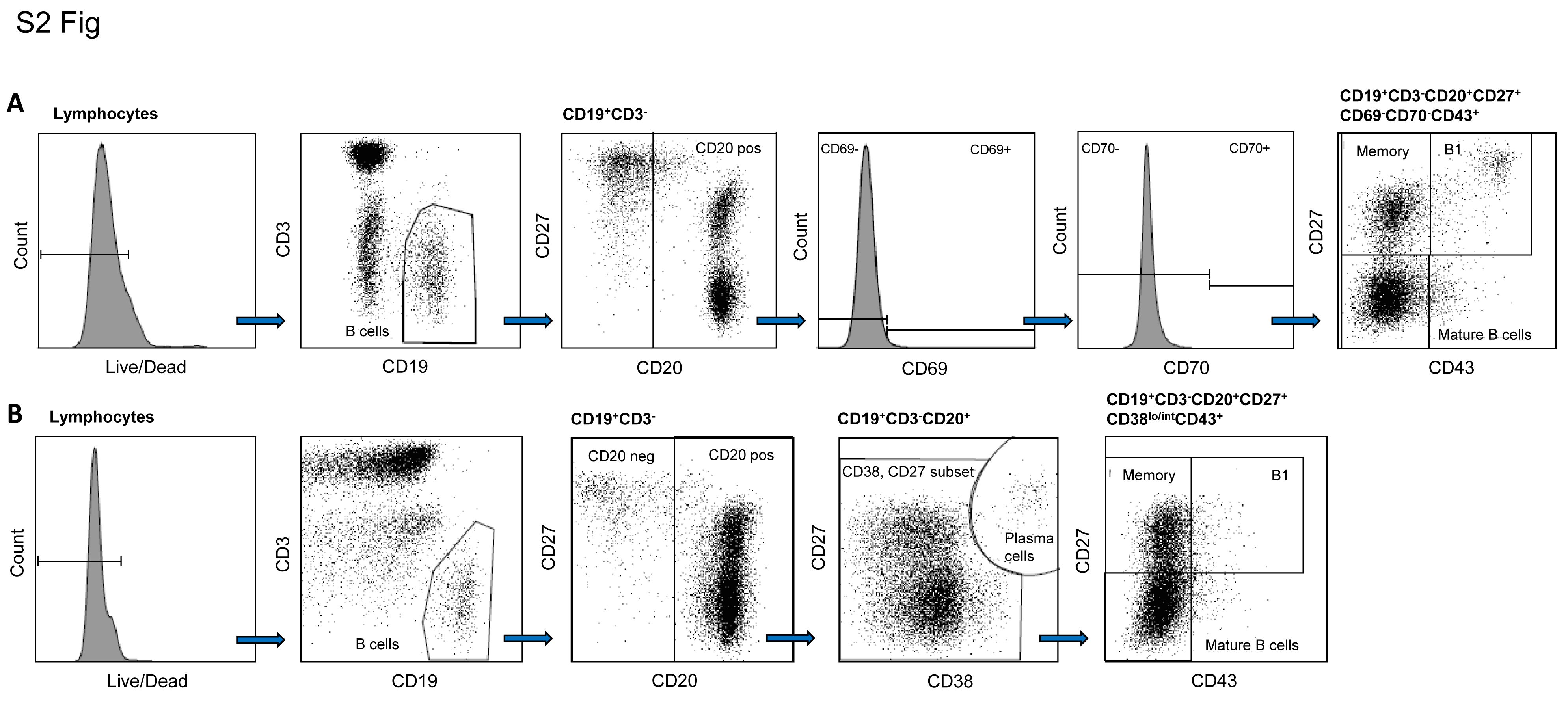

Supplement: S2 Fig — Flow cytometry was performed on a three-laser FACSCantoII and analyzed with FlowJo Software 10.0.8r1. (TIF) [file pone.0152215.s002.tif]

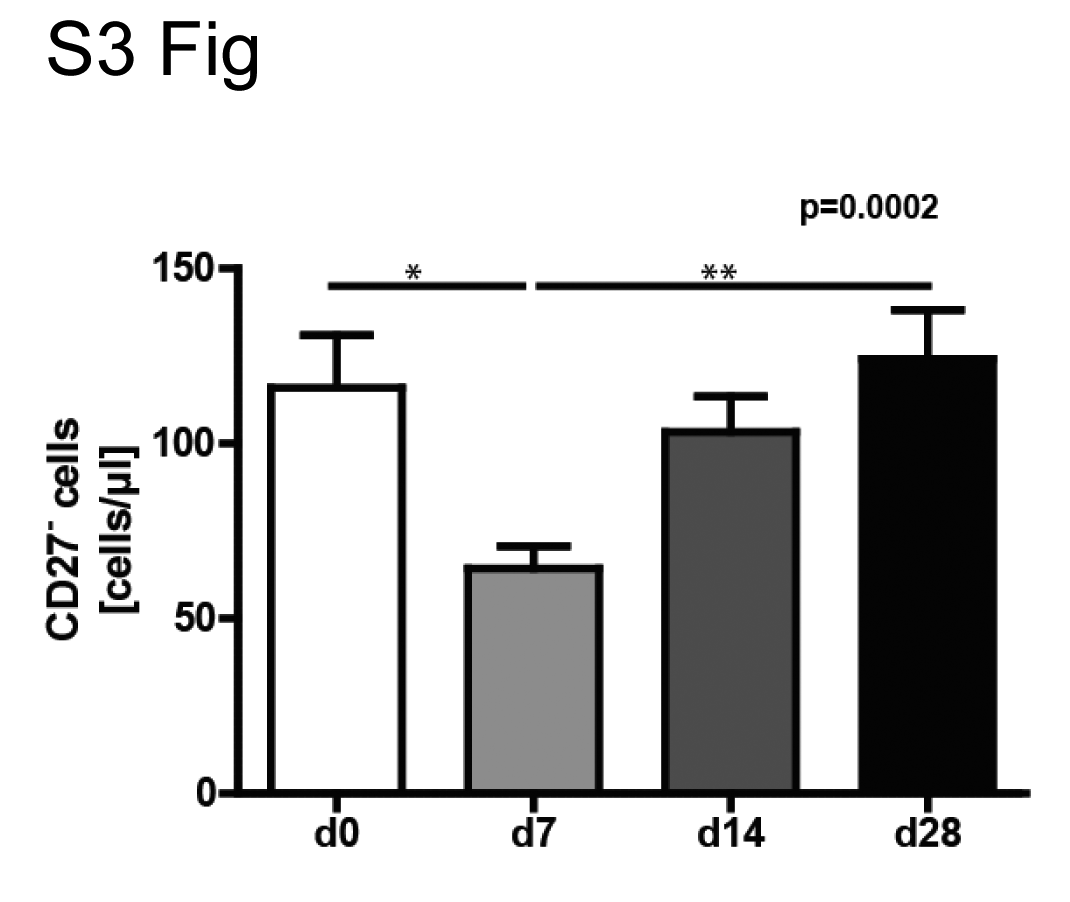

Supplement: S3 Fig — Absolute numbers of CD27- B cells after immunization. Mean values ± SEM are indicated for each time point (*p<0.05 **p<0.01). (TIF) [file pone.0152215.s003.tif]

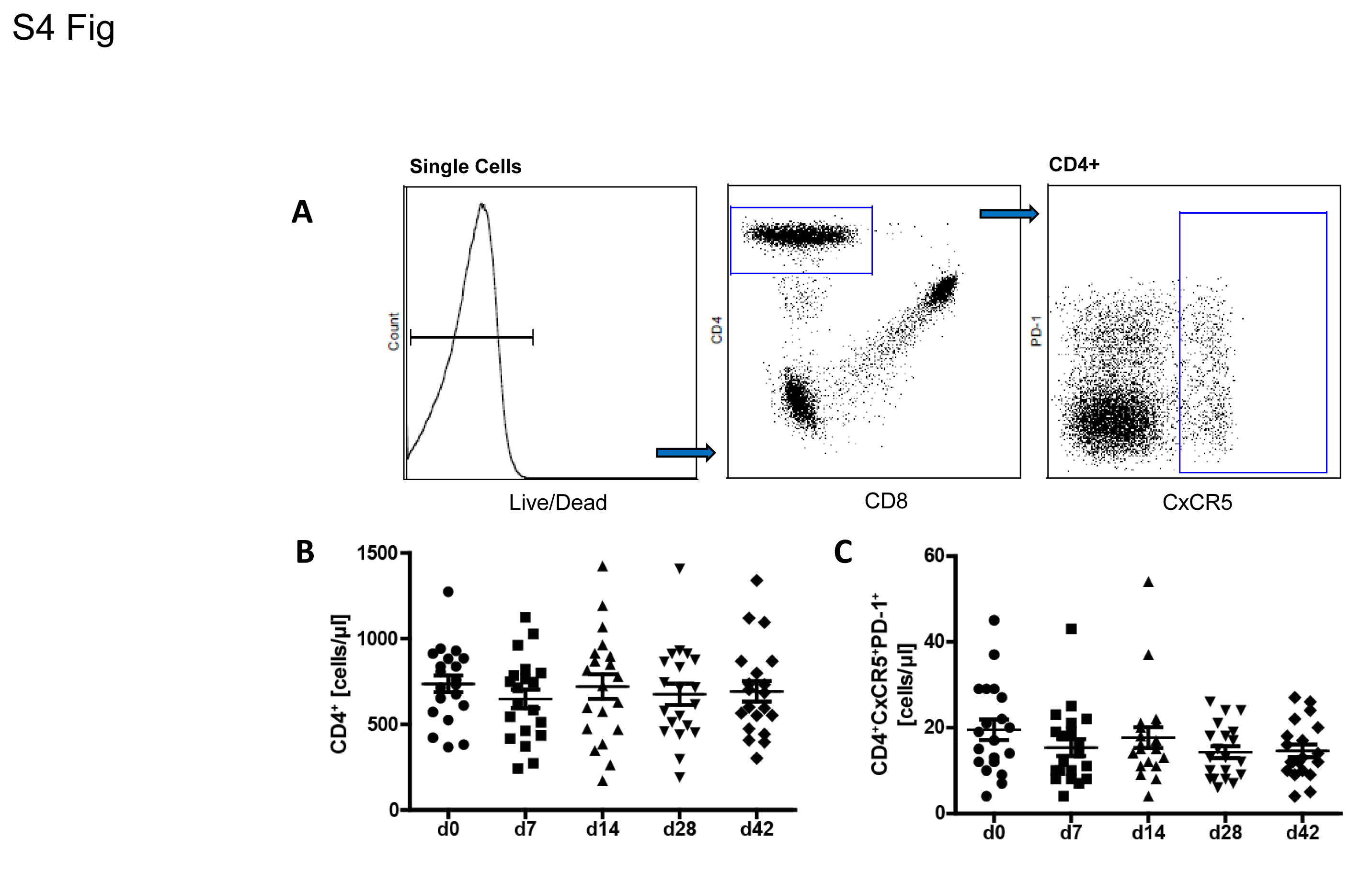

Supplement: S4 Fig — (A) Gating strategy of CD4+CxCR5+PD-1+ TFH cells with absolute numbers of (B) CD4+ T cells and (C) CD4+CxCR5+PD-1+ TFH cells after immunization (n = 20). (TIF) [file pone.0152215.s004.tif]
